# Simultaneous assessment of spontaneous cage activity and voluntary wheel running in group-housed mice

**DOI:** 10.1038/s41598-022-08349-z

**Published:** 2022-03-15

**Authors:** Annika Reuser, Kristin Wenzel, Stephan B. Felix, Marcus Dörr, Martin Bahls, Stephanie Könemann

**Affiliations:** 1 Department of Internal Medicine B, University Medicine Greifswald, Fleischmannstr. 41, 17489 Greifswald, Germany; 2 German Centre of Cardiovascular Research (DZHK), Partner Site Greifswald, Greifswald, Germany

**Keywords:** Preclinical research, Cardiovascular biology, Circulation, Metabolism

## Abstract

Small animal models are frequently used to improve our understanding of the molecular and biological signaling pathways underlying the beneficial effects of physical activity and exercise. Unfortunately, when running wheels are employed, mice and rats are often kept single-housed to determine the individual running distance of each animal. However, social isolation can be stressful for rodents, and may alter an individual’s propensity for or response to exercise. For example, increased stress from single housing may significantly affect the results when investigating systemic metabolic responses to exercise. We have combined two already available and well-established systems, a radiotelemetry system and a running wheel, to determine spontaneous cage activity (SCA) as well as voluntary exercise (VE) levels of the individual animal in group-housed rodents. Further, we developed a simple software tool which allows monitoring and analyzing the data. Specifically, the radiotelemetry-system utilizes radio-frequency identification via a small, implanted chip to determine the location of each animal. Since, in addition to the animals’ position, also the location of the running wheel in the cage is known, the conclusion of which animal is exercising can be drawn. The developed software enables a fast and reliable assignment of the VE data to the individual animal and a simple analysis of the data collected. Hence, our combined method may be used to investigate the beneficial effects of physical activity, as well as the impact of therapeutic interventions on animal behavior in group-housed rodents.

## Introduction

Regular exercise and physical activity are hallmarks of a healthy lifestyle. Further, exercise reduces the risk for cardiovascular morbidity^1^ and mortality^2^. To improve our understanding of the underlying biology animal models are frequently used. However, assessing spontaneous cage activity (SCA) in combination with voluntary running wheel exercise (VE) in rodents can be difficult especially under consideration of a species-appropriate husbandry^3^.

A wide range of long-term SCA tracking systems like video-tracking^4^, infrared-based systems^5^, or force-plate actometers^6^ (with a variety of additional features) are already available. Each method allows researchers to collect information about the animals’ movement patterns, but require that animals must be kept in species-inappropriate husbandry due to single-housing or 24-h lighting. This is thought to strongly influence the animals’ SCA^7^ and is seen critically in Western animal welfare laws requiring animals to be housed according to their specific needs. Although some methods allow group-housing, they often demand invasive or recurrent procedures, e.g. cutting off the tail or using markers which may influence the animals’ organism by soaking up the ingredients through the skin or the mouth. We are unaware of any established SCA tracking systems which simultaneously measure VE on an individual level in group-housed animals.

Here we report an evolved method, combining a validated and well-established radiotelemetry system and an already available running wheel, to measure SCA and VE of each animal individually without the need for single-housing or debilitating labelling. The radiotelemetry system is based on an electromagnetic field in which the movement patterns of each animal equipped with a radio frequency identification device (RFID) can be detected. To measure VE we integrated a running wheel into the animals’ cage. Since the location of the wheel in the cage is known, VE can be assigned to an individual animal as we know the animals’ positions at the time point the VE was measured by analyzing the SCA data. To warrant easy and reliable assignment and quick data analysis, we developed a new software termed SCAVE (Spontaneous Cage Activity and Voluntary Exercise). This combination and the developed software enable the researcher to easily track animals’ individual movement and activity patterns without affecting its natural behavior by single-housing, recurrent labeling, or surgical procedures.

## Materials and methods

### Animals

For the experiments female transgenic αMHC Gαq mice with a Friend leukemia virus strain B (FVB) background were used at the age of 5 to 6 weeks. The strain was shown to develop cardiac hypertrophy in response to overexpression of Gαq^8^. Data were gathered over a period of 7 weeks, wherein three to four animals were housed in a cage. We used “Randomization in Treatment Arms” (RiTa; https://www.evidat.com/rita) to randomize the mice (n = 13) into two groups: control (CON; n = 7) and exercise (EX; n = 6). In both groups SCA was tracked by a radio telemetry system. Only group EX had access to a running wheel. To minimize confounders the cage with the tracking system was left at the same position during the experiments. The mice were maintained on a 12-h dark–light cycle (changing at 6 a.m. and 6 p.m.) and fed ad libitum standard animal chow. Animal experiments were performed in accordance with the German animal protection act. Permission for the conduction of the animal experiments was obtained from the committee on animal welfare of the federal state of Mecklenburg-Western Pomerania, Germany (registry-no. 7221.3–1–054-16). All authors complied with the ARRIVE guidelines.

### Radio telemetry tracking system

**Figure 1:**
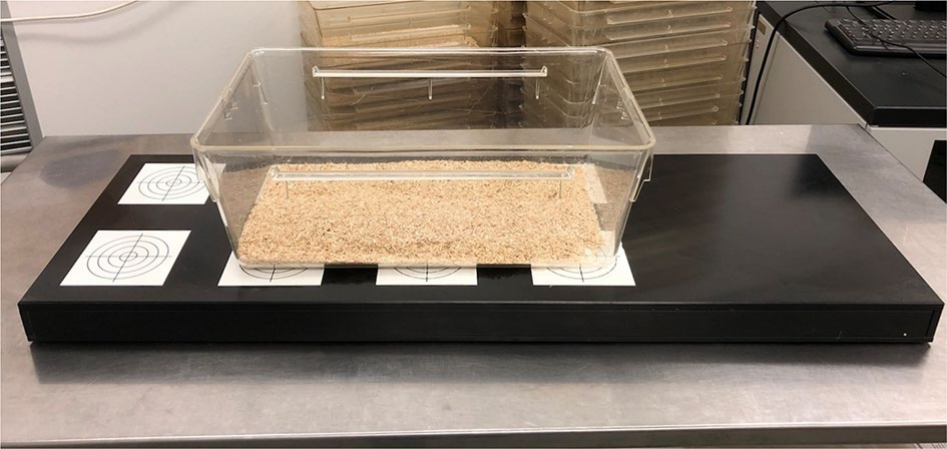
Overview of the radio telemetry tracking system. The radio telemetry platform is made up of eight antennas generating electromagnetic fields. A polycarbonate cage is placed on top of it.

For SCA acquisition, a radiotelemetry system was used (TSE TraffiCage; model: Type III No 1290D; TSE Systems, Bad Homburg, Germany, Fig. 1). This system consists of a long, flat platform (425 × 266 × 155 mm) connected to a standard-PC (Microsoft, Windows 7 Professional (2011); 64-Bit-Software). The radio telemetry platform is made up of eight same sized antennas arranged in two rows to generate eight equally sized electromagnetic fields (95 × 95 mm). Every antenna detects the presence or absence of a RFID-chip within its own electromagnetic field, using a sampling frequency pre-defined by the operator. Temporal patterns of transponder readings are conveyed immediately to a customized software (TraffiCage, TSE Systems, Bad Homburg, Germany) installed on the connected PC. The software records the presence or absence of a RFID-chip within the different antenna areas with time stamps. Hence, we have information on where each animal was at any given point in time with a precision of a few milliseconds (manufacturer's instruction, TSE Systems, Bad Homburg, Germany). Further, the data must be exported to Excel, MATLAB or other spreadsheet programs for reading and analysis.

A standard laboratory polycarbonate cage (290 × 220 × 140 mm, 4 mm ± 0.5 mm thick) was placed on top of the electromagnetic platform whereby it extended over a size of about two by three antennas. To track the animals’ position, each mouse was equipped with a RFID-chip.

### RFID transponder injection

**Figure 2:**
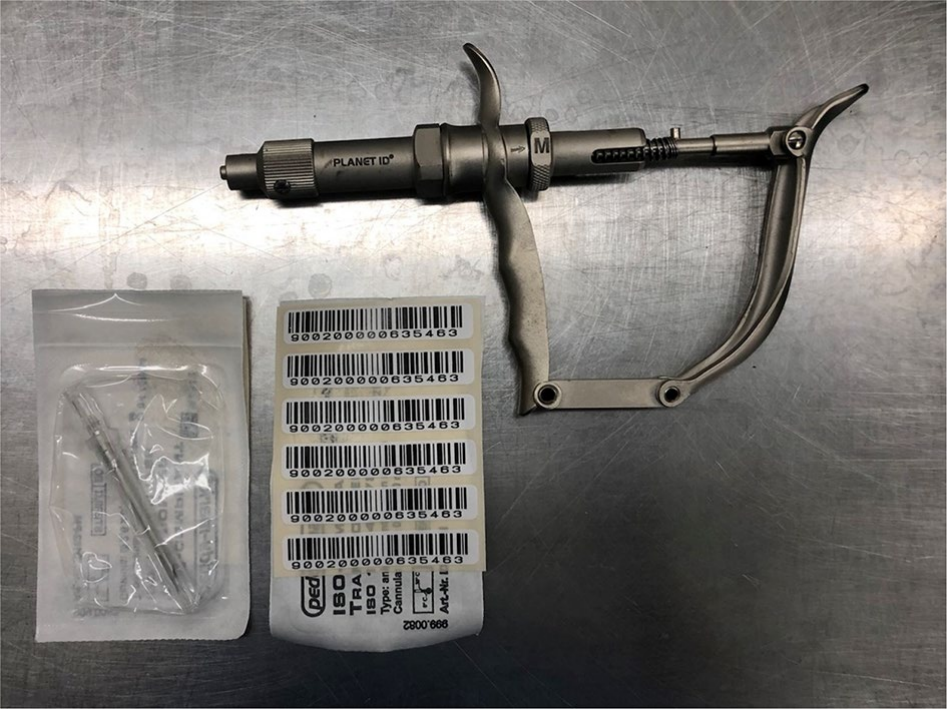
RFID Injection gun. Injection gun with a special needle containing the coded RFID which is implanted in the mice’ subcutis.

For the RFID implantation an injection gun was used (Fig. 2). The penetration depth could be varied (short (S), medium (M), or large (L) penetration depth) depending on the animal’s size and weight. The transponder was implanted at the level of the scapulae by taking up a fold of skin with two fingers and inserting the chip immediately below. The implantation was performed under anesthesia using short-acting isoflurane to protect the animal from injuries caused by its own movements.

### Monitoring VE

For exercise measurements a running wheel (TSE Systems, Bad Homburg, Germany) was placed in the cage and connected to a computer. A rotary sensor integrated into the wheel measures wheel revolutions, the movement direction, as well as the beginning and end of the movement. Since the running wheel is not equipped with a RFID-antenna, the VE that all animals in the cage ran in total is measured. Those data were automatically transferred to a customized software (RunningWheel; TSE Systems, Bad Homburg, Germany), which calculated average and maximum speed in rotations per minute, as well as the temporal length, in seconds, finally, the data had to be exported to Excel, MATLAB, or other spreadsheet programs for analysis.

### Assessment of SCA data

**Figure 3:**
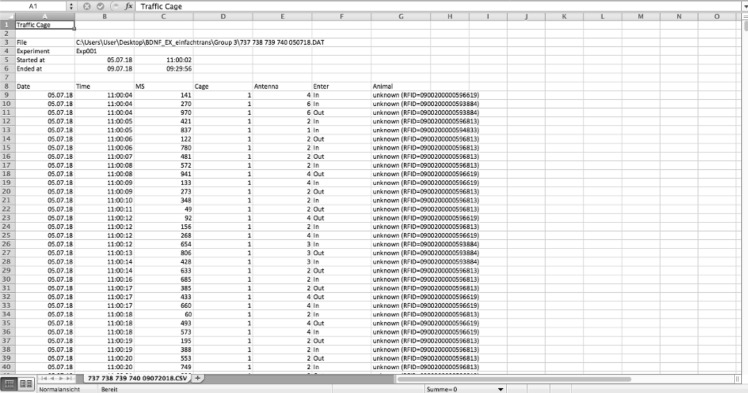
SCA data exported to a spreadsheet program. It is noted at which point of time (“Date”; “Time”) which animal (“ANIMAL”—“RFID number”) entered or left which antenna (“Cage”; “Antenna”; “Enter”—“IN”; “ENTER”—“OUT”) and how long it stayed there (“MS”).

**Figure 4:**
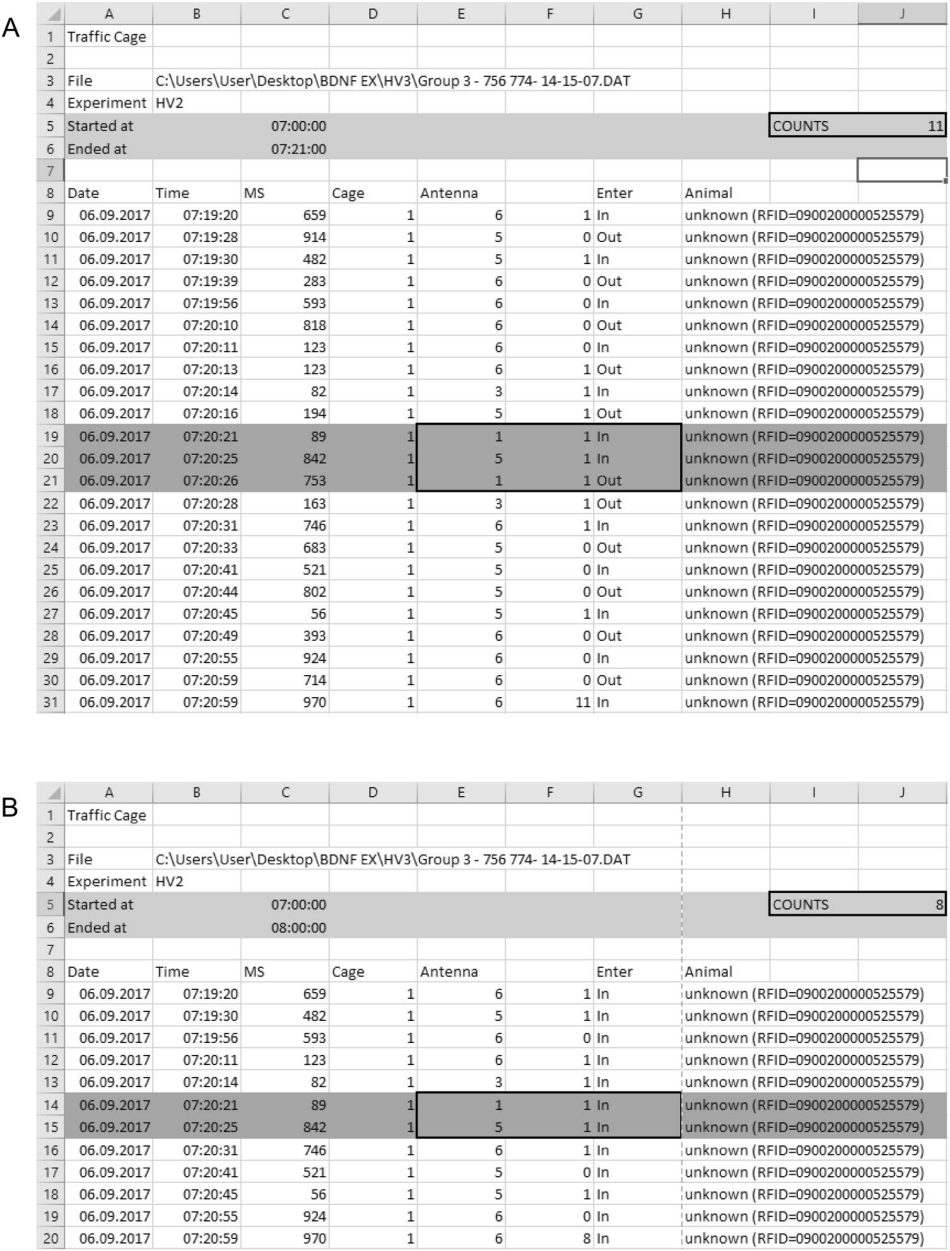
Doublings can be avoided by eliminating the registration of leaving an antenna. Excel compares each position of the animal in the column with the following position recorded. Depending on whether the registering antenna has changed or not between two measurement points, Excel notes a one or a zero in the next column. (**A**) By summing up all entries, the user receives the number of field changes over a chosen time period. (**B**) Using this method, Excel frequently counts more field changes than the animal actually performed.

The SCA data is displayed as tables of the different spreadsheet programs and is sorted by timestamps (Figs. 3, 4). Although the radio telemetry platform detects the animals’ position several times a second, only those timestamps that are associated with a change in antenna field are listed. Thus, in every line it is noted which field (“Antenna”—“1–6”) was entered (“Enter”—“IN”) or left (“ENTER”—“OUT”) by which animal (“ANIMAL”—“RFID number”). Additionally, the calculated time the animal spent on the antenna (“MS”) as well as the cage number is noted (“CAGE”).

### Assessment of VE data

**Figure 5:**
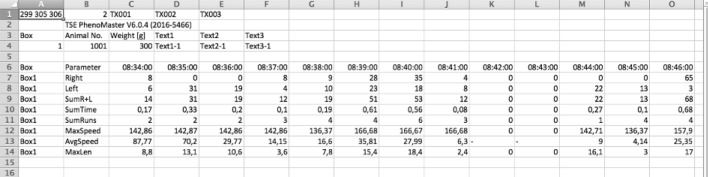
VE data exported to a spreadsheet program. Every minute the wheel rotations are noted (“Right” rotations to the right; “Left” rotations to the left; “Sum R + L” the sum of rotations to the right and to the left) as well as the duration of the activity (“Sum Time”) and the number of runs (“Sum Runs”). Additionally, maximum speed, average speed and the length of activity is calculated (“MaxSpeed”; “AvgSpeed”; MaxLen”).

The VE data was exported as tables, just like the SCA data (Fig. 5). However, VE cannot be directly attributed to an individual animal, as the running wheel cannot detect the RFID. Instead, it must be assigned to the individual by matching the VE data to the SCA data. As the SCA information indicates which animal entered (and at which specific point in time) the antenna that the running wheel was placed on, the conclusion of which animal exercised can be drawn. The SCAVE analyzing tool can be used to make this assignment.

### SCA and VE analyzing tool (SCAVE)

**Figure 6:**
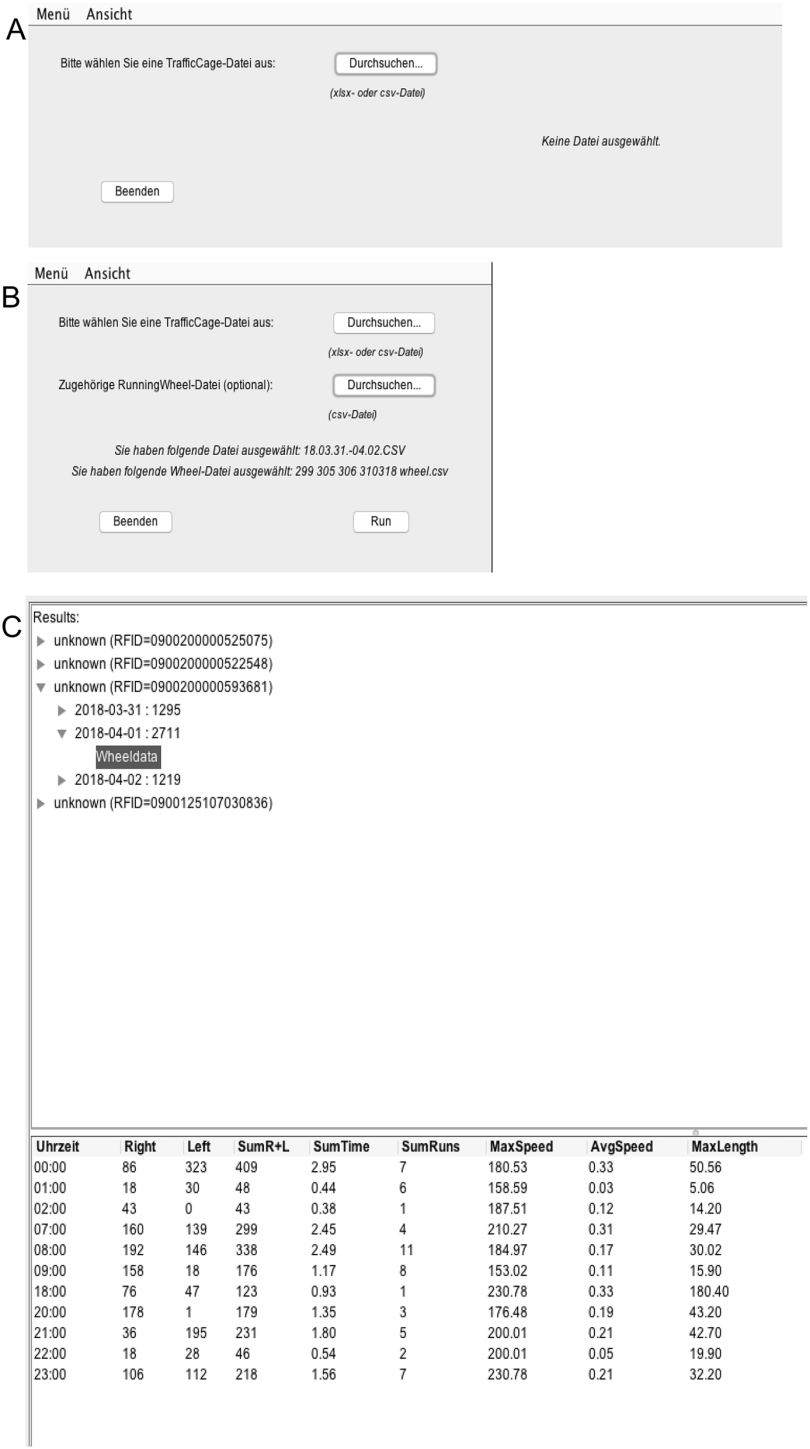
Overview of the SCAVE software (Version 2.0; Appendix 1). (**A**) Opening the software, the user can open a SCA file alone or in combination with the corresponding VE file, (**B**) both in cvs. format. By pressing “RUN” the software (**C**) calculates the results for each animal.

We developed the software SCAVE (Spontaneous Cage Activity and Voluntary Exercise measurement; Version 2.0; Appendix 1) to quickly, easily, and reliably analyze the SCA spreadsheet data, and calculate VE parameters for each animal by integrating the SCA data with the running wheel data (Fig. 6A,B). The only premise for our software is that the running wheel must be placed on antenna 1 and 2. SCAVE assigns the number of detected wheel rotations at a specific time point to the animal that (a) entered antenna 1, antenna 2 or both antennas on which the running wheel was placed and (b) was not detected by the radiotelemetry system during the time the wheel rotates since animals exercising in the wheel cannot be detected by the TraffiCage due to distance from the antenna and the running wheel’s material. If VE was measured but no animal fulfills both premises, the measurement at this time point is discarded. Since the software relies on the TraffiCage data for assigning VE to the individual animal, the running wheel must be placed correctly on the TraffiCage platform on antenna 1 and 2. Meeting these criteria is very important to avoid incorrect assignments. Below are three scenarios users may encounter.

**Figure 7:**
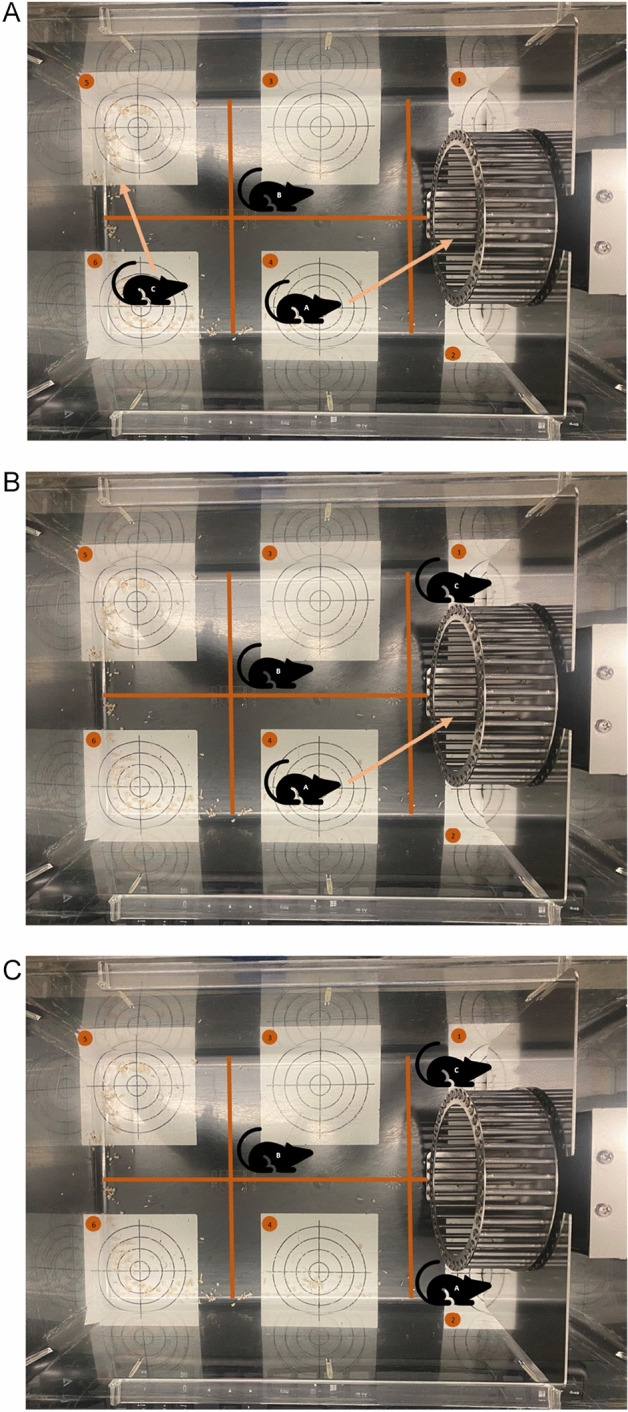
Different scenarios that show how the SCAVE software works. (**A**) A single animal is exercising while the other two are spread over the cage. (**B**) A single animal is exercising while one is sitting next to wheel. (**C**) Two animals are sitting next to the running wheel and are moving it.

Scenario 1: A single animal is exercising while the other two are spread over the cage (Fig. 7A): First, animal A is resting on antenna 4, then crosses antenna 2 to enter the running wheel. Animal B is resting on antenna 3, Animal C on antenna 6 changing to antenna 5. Only animal A fulfills both premises (a) entering one of the antennas the running wheel is placed on (in this case antenna 2) and (b) is not detected by the radiotelemetry system during wheel rotations are counted (which occurs as soon as an animal enters the wheel as described above). According to this the VE is assigned to animal A.

Scenario 2: A single animal is exercising while one is sitting next to wheel (Fig. 7B): First, animal A is resting on antenna 4, then crosses antenna 2 to enter the running wheel. Animal B is resting on antenna 3. Animal C is resting on and thus detected by antenna 1. Accordingly, animal C would meet criteria (a) (entering one of the antennas the running wheel is placed on: in this case animal C is detected by antenna 1) but not criteria (b) (not being detected while VE is measured: animal C detected by antenna 1 further on since it did not enter the running wheel). Again, only animal A fulfills both premises. According to this, the VE is assigned to animal A and a mismatch to animal C is avoided.

Scenario 3: Two animals are sitting next to the running wheel and are moving it (Fig. 7C): animal A is sitting on antenna 2, animal C on antenna 1. One of them is moving the wheel from the outside e.g. by using its paws. Animal B is resting on antenna 3. Wheel rotations are counted but discarded because none of the animals fulfills both premises. Animals A and C meet criteria (a) (entering one of the antennas the running wheel is placed on: in this case antenna 1 and 2) but none of them meets criteria (b) (not being detected while VE is measured: animals A and C are still detected by antenna 1 and 2). Accordingly, VE is discarded. Further reasons for measured VE not matching both criteria could be artificial wheel rotations caused by humans cleaning or moving the cage or weighing the animals.

Depending on the animals’ size it is possible that two animals are exercising simultaneously. Since both fulfill premise (a) as well as premise (b) the VE is assigned to each animal.

The software displays the analyzed data in an individual table for each animal showing the SCA data per animal per hour either alone or in combination with the VE data per animal per hour (Fig. 6C). The software SCAVE Version 2.0 is licensed as CC 4.0 and can be downloaded at: https://figshare.com/s/7bdc5d2ee9a5be8548c5 (download and software instruction Appendix 1).

### Sensitivity assessment

We assessed the ability of our system to detect changes in physical activity in a transgenic mouse model of hypertrophy. SCA and running wheel distance were measured over a period of 7 weeks.

### Statistical analysis

All values are expressed as mean ± SEM. To investigate the influence of voluntary wheel running on spontaneous cage activity a one-way ANOVA was used. Statistical significance was defined as p < 0.05.

## Results

### SCA per week

**Figure 8:**
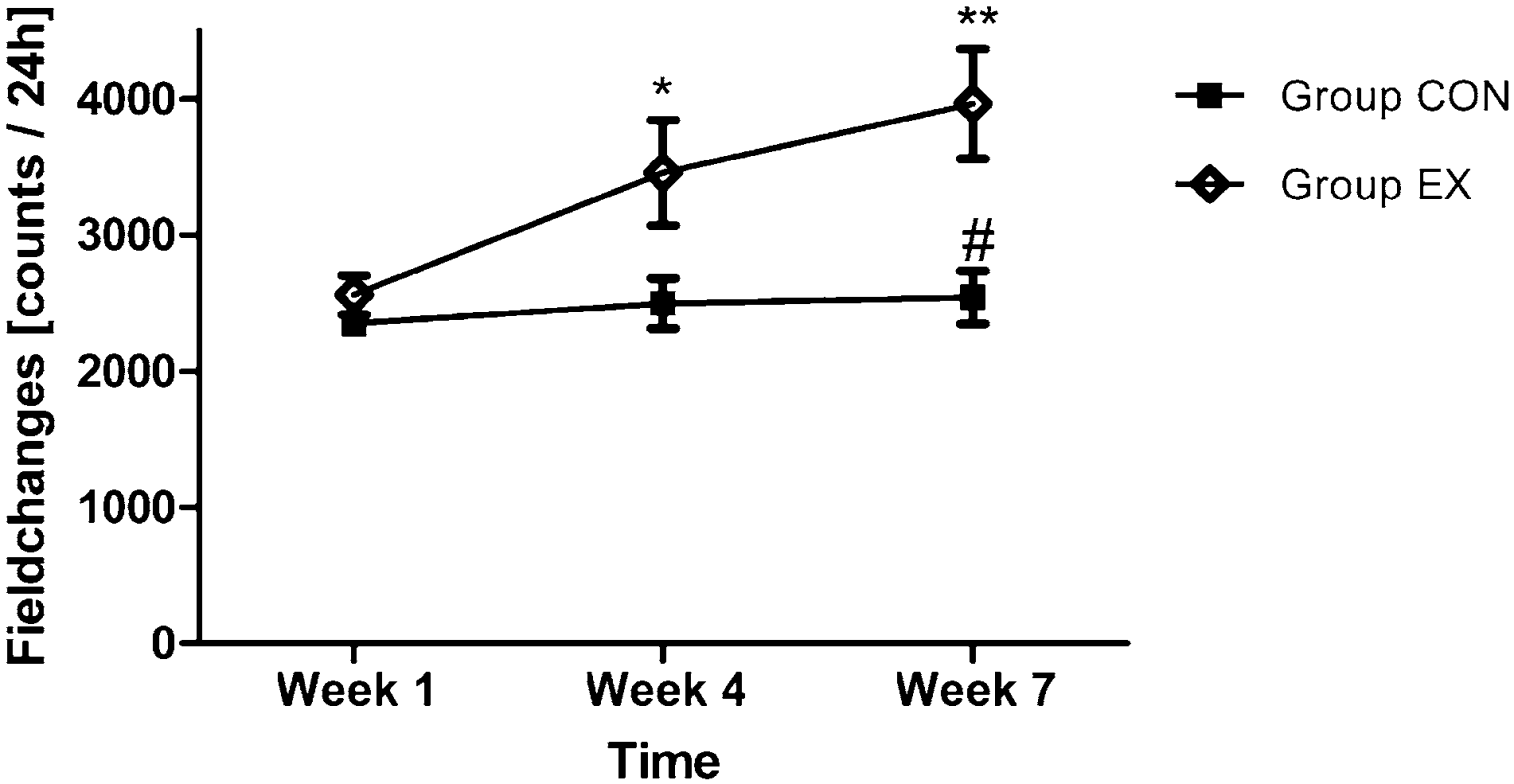
Field changes [counts] per 24 h Week 1 vs. Week 4 vs. Week 7. The field changes in the control group (Group CON) remained constant over the study period of 7 weeks. Spontaneous cage activity (SCA) significantly increased within the exercise group (Group EX) during the experimental period (*p < 0.05 and **p < 0.005 vs. Week 1) and was significantly higher than the SCA of the Group CON (^#^p < 0.05 vs. Group CON same week).

The CON and EX group started with similar SCA (CON: 2351.64 field changes per 24 h (fc/24 h); EX: 2561.98 fc/24 h; Fig. 8). While the field changes of the CON group remained constant over the study period of 7 weeks (2498.71 fc/24 h week 4; 2544.63 fc/24 h week 7; Fig. 8), SCA significantly increased within the EX group during the experimental period (3458.66 fc/24 h week 4; 3964.99 fc/24 h week 7; p = 0.03 week 1 vs. week 4; p = 0.001 week 1 vs. week 7; Fig. 8) and was significantly higher than the SCA of the CON group at week 7 (p = 0.03; Fig. 8).

### SCA per day

**Figure 9:**
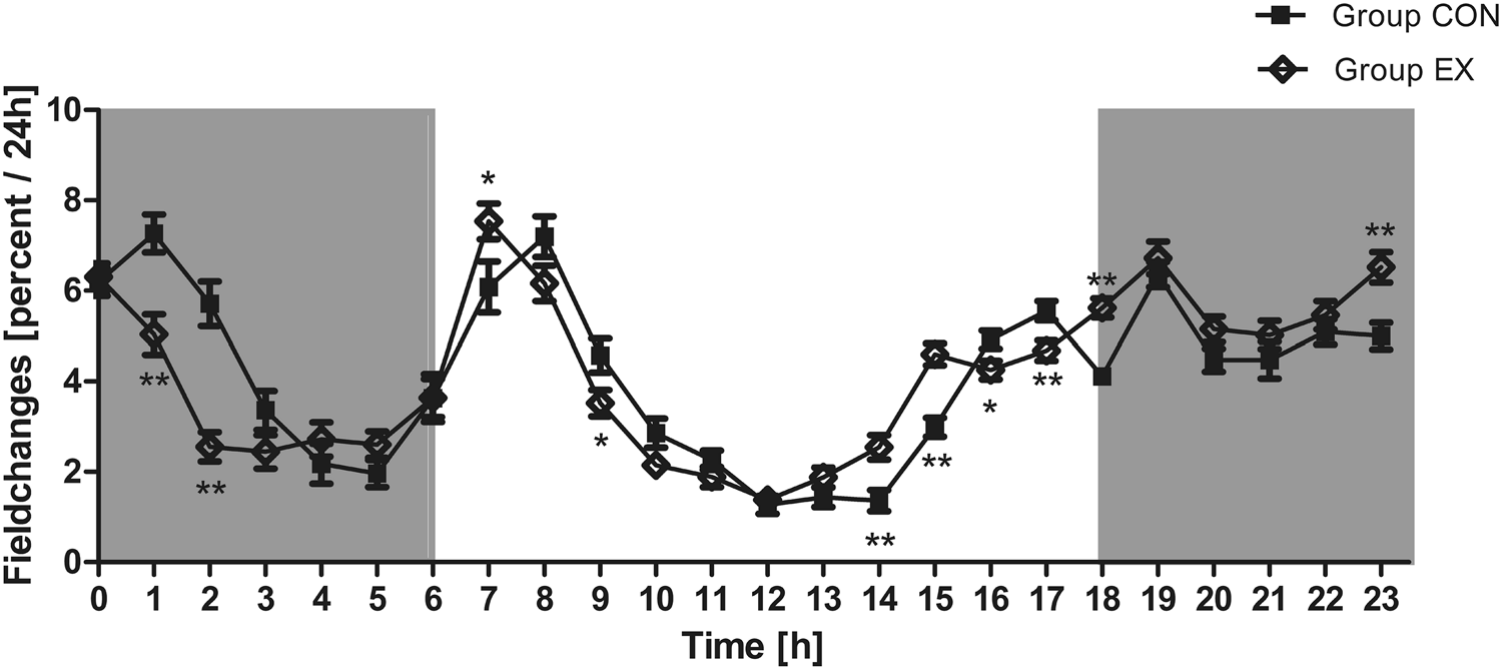
Field changes [percent] per hour. The control groups’ (Group CON) and exercise groups’ (Group EX) daily movement patterns were similar with some significant deviations (Group CON vs. Group EX at the time *p < 0.05 and **p < 0.005).

Although the two groups showed major differences regarding their SCA, the mice’ daily movement patterns were similar with only some significant deviations. Overall, both groups showed the highest voluntary cage activity during the dark phase while they were mostly inactive during the light period. SCA reached its highest plateau from 8 p.m. to 12 a.m. with a stable activity of about 4–7% of the total daily activity (average field changes per 24 h: 8 p.m. 4.81%; 9 p.m. 4.75%; 10 p.m. 4.78%; 11 p.m. 5.76%; 12 a.m. 6.25%; Fig. 9) and declines thereafter. In contrast, the lowest activity levels were recorded from 10 a.m. to 1 p.m. with about 1–3% of the total daily activity (average CON and EX field changes per 24 h: 10am 2.5%; 11 a.m. 2.08%; 12 a.m. 1.33%; 1 pm 1.66%; Fig. 9) followed by an increase in the afternoon. In both groups the SCA levels peak sharply when the lights were turned on (CON 8 a.m. 7.19%; EX 7 a.m. 7.54%; Fig. 9) and off (CON 7 p.m. 6.35%; EX 7 p.m. 6.72%; Fig. 9) and decreased subsequently. Significant differences between the two groups are caused by an earlier decline and an increase in the activity levels of the EX group in contrast to the CON group (e.g. at 2 a.m. CON 2 a.m. 1.36%; EX 2 a.m. 2.54%; p = 0.0015; Fig. 9).

## Discussion

Here we introduce an evolved method to simultaneously monitor SCA and VE of individual animals in group-housed mice combining two well-established systems. Further, we developed a software that works reliably and allows simple and quick data analysis. Both may be implemented in any study that aims to investigate individual movement patterns of group-housed mice with or without VE under different treatment-conditions.

Since we combined two already established systems, we compared the measured SCA with previously published data of other groups to validate our results^9,10^. Our SCA results show that mice were less active during the light phase, apart from the noticeable peak activity from seven am to eight am. This was most likely due to staff starting to clean the rooms and cages at this time. However, activity consistently increased during the afternoon. During the first hours of the dark phase our mice reached the highest activity levels followed by an abrupt and strong activity drop in the ensuing hours until activity approached those measured during the light phase. In comparison, Malisch et al. measured cage activity in mice using force plate actometers, Dudek et al. also used the TraffiCage system to measure SCA in obese rats^9^. Overall, their findings are similar to ours with the exception that especially Malisch et al. found constantly low cage activity levels during the light phase^11^. However, this may be explained by disturbances due to cage cleaning, water bottle, and food changes several times a week in our laboratory. Slight discrepancies in the dark–light movement patterns, like the increase of activity during the light phase or the contrast between the activity levels during light and dark that are not as distinctive as those measured by Malisch et al., could be attributed to the animals’ genetic background. FVB mice show a more fragmented and arrhythmic activity pattern with increased activity during the light phase due to retinal degeneration^12^. Consequently, our mice show SCA patterns during the dark phase which were similar to those recorded by Malisch et al. and Dudek et al., and the movement patterns during the light phase deviate as expected.

We acknowledge that our method has some limitations. For example, our system is not able to detect special behavioral patterns or distances covered by the animal like other instruments do. Instead, field-changes are used as a surrogate for SCA. Physical activity which takes place within the field of one antenna cannot be measured. Due to the small size of the antennas (9.5 × 9.5 cm) relevant physical activity, like running in circles, leads to crossing the antenna’s border and hence being measured. Furthermore, by summing up all field changes over a defined period, it frequently happens that more field changes are counted than the animal actually performed, as both the animal’s entry of and exit from an antenna field is noted in the table. As an animal is entering the area of an antenna before it leaves the previous antenna, an additional field change is counted incorrectly (Fig. 4A,B). With the use of our analyzing tool SCAVE (Version 2.0; Appendix 1) the registration of the incorrect exits of an antenna area is eliminated and doublings can be avoided. Further, the RFID needs to be injected subcutaneously which requires our mice to be anesthetized. Despite these limitations, radio telemetry activity tracking is a reliable, good-working alternative to other established methods as it allows species-appropriate husbandry according to Western animal welfare laws. The usage of our newly developed software SCAVE ensures a fast and reliable analysis of SCA as well as VE data. While the animals’ chipping is still minimally invasive, it enables the monitoring without extensive surgeries or recurrent marking with potentially harmful substances. In addition, radio telemetry allows 12-h dark–light cycles as well as experiments that require other day/night-cycles. Taken together, our combined approach enables simultaneous SCA and VE measurements and provides new possibilities to assess the impact of voluntary wheel running on cage activity as well as the influence of therapeutics on animal behavior.

## Supplementary Information

Supplementary Information.
