# Publisher Correction: Simultaneous assessment of spontaneous cage activity and voluntary wheel running in group-housed mice

**DOI:** 10.1038/s41598-022-12160-1

**Published:** 2022-05-12

**Authors:** Annika Reuser, Kristin Wenzel, Stephan B. Felix, Marcus Dörr, Martin Bahls, Stephanie Könemann

**Affiliations:** 1 Department of Internal Medicine B, University Medicine Greifswald, Fleischmannstr. 41, 17489 Greifswald, Germany; 2 German Centre of Cardiovascular Research (DZHK), Partner Site Greifswald, Greifswald, Germany

Correction to: *Scientific Reports* https://doi.org/10.1038/s41598-022-08349-z, published online 15 March 2022

In the original version of this Article, Annika Reuser was incorrectly listed as a corresponding author. The correct corresponding author for this Article is Martin Bahls. Correspondence and request for materials should be addressed to martin.bahls@uni-greifswald.de.

The original Article has been corrected.

