# Supplementary material for: Simultaneous assessment of spontaneous cage activity and voluntary wheel running in group-housed mice
Source: Sci Rep. 2022 Mar 15;12:4444. doi: 10.1038/s41598-022-08349-z (PMC8924253; doi:10.1038/s41598-022-08349-z)
Supplement: Supplementary file 1 — Supplementary Information. [file 41598_2022_8349_MOESM1_ESM.doc]

**Appendix 1**

User Guide

1. Download and install Java (Version 8 - April, 17th 2018 or later). Free download for Windows, Mac OS, Linux, etc.
2. Download and install the program „SCAVE 2.0“ (private link: https://figshare.com/s/7bdc5d2ee9a5be8548c5).
3. Open „SCAVE 2.0“.
4. Press „Durchsuchen“ on the user interface to select a data file in xls-format recorded by the TraffiCage.
5. Optional, the corresponding RunningWheel file in xls-format can be selected by pressing „Durchsuchen“.
6. Press „Run“ to analyze the data sets.
7. A new interface appears listing the mice’ RFID-chip-numbers.
   1. Choose a mouse / a RFID-chip by double-clicking.
   2. Select a date by clicking 🡪 the analyzed data are listed in table format.
   3. If a RunningWheel file was chosen additionally, click on the arrow on the left side of the selected date to open the analyzed RunningWheel data.
